# Treatment of glioblastoma with herbal medicines

**DOI:** 10.1186/s12957-018-1329-2

**Published:** 2018-02-13

**Authors:** Ivo Trogrlić, Dragan Trogrlić, Darko Trogrlić, Amina Kadrić Trogrlić

**Affiliations:** Family business “DREN” Ltd, Žepče, Bosnia and Herzegovina

**Keywords:** Phytotherapy, Glioblastoma, Recurrence, Nuclear magnetic resonance, Computed tomography

## Abstract

**Background:**

In the latest years, a lot of research studies regarding the usage of active agents from plants in the treatment of tumors have been published, but there is no data about successful usage of herbal remedies in the treatment of glioblastoma in humans.

**Methods:**

The phytotherapy involved five types of herbal medicine which the subjects took in the form of tea, each type once a day at regular intervals. Three patients took herbal medicine along with standard oncological treatment, while two patients applied for phytotherapy after completing medical treatment. The composition of herbal medicine was modified when necessary, which depended on the results of the control scans using the nuclear magnetic resonance technique and/or computed tomography.

**Results:**

Forty-eight months after the introduction of phytotherapy, there were no clinical or radiological signs of the disease, in three patients; in one patient, the tumor was reduced and his condition was stable, and one patient lived for 48 months in spite of a large primary tumor and a massive recurrence, which developed after the treatment had been completed.

**Conclusions:**

The results achieved in patients in whom tumor regression occurred exclusively through the use of phytotherapy deserve special attention.

In order to treat glioblastoma more effectively, it is necessary to develop innovative therapeutic strategies and medicines that should not be limited only to the field of conventional medicine. The results presented in this research paper are encouraging and serve as a good basis for further research on the possibilities of phytotherapy in the treatment of glioblastoma.

## Background

Glioblastoma multiforme falls into the group of astrocytic tumors. It is a most malignant intracranial tumor, and according to the classification by the World Health Organization (WHO), its degree of differentiation is IV [(GBM) grade IV] [1]. According to the manner of their formation, glioblastoma multiformes (GBMs) are divided into primary GBMs, occurring de novo and accounting for about 90% of all GMBs, and secondary GBMs, which occur due to a malignant progression of lower grade astrocytoma. While in well-differentiated pilocytic astrocytoma (WHO grade I), only rare cases of malignant progression have been recorded, progression to a higher grade is almost the rule in diffuse (WHO grade II) and anaplastic (WHO grade III) astrocytoma. In diffuse astrocytoma, the average time to progression into GBM is approximately 5 years, while anaplastic astrocytoma takes half the time for malignant progression [2]. Although secondary GBMs have a slightly better prognosis than the primary types, most patients with diffuse and anaplastic astrocytoma, where there is a malignant progression, die within a year after the progression into GBM [3].

Current results achieved in the treatment of GBM are unsatisfactory. The median survival is from 5 months for the patients with primary GBMs to 8 months for those with the secondary GBMs, while a five-year survival is achieved only in about 2% of patients [4, 5]. In the past 15 years, the greatest breakthrough in the treatment of GBM has been achieved by the introduction of an alkylating agent temozolomide (TMZ) which, together with radiotherapy followed by monotherapy in 6 cycles of 28 days, prolongs the life of some patients (Stupp protocol-Stupp regimen) [6]. However, even this method of treatment does not provide patients a longer disease-free period of time, because in patients who responded well to this therapy, the recurrence occurs within 7 months on average [7].

The use of TMZ in the treatment of GBM is limited by the activity of the gene MGMT (O6-methylguanine-DNA methyltransferase) in tumor cells. This gene encodes an enzyme that effectively repairs the damage caused by TMZ and other alkylating cytostatics used in the treatment of tumors, which significantly reduces their effectiveness [8].

The epigenetic silencing of tumor suppressor genes by the methylation of their promoter is an early event in carcinogenesis, and it leads to the inactivation of these genes, which opens the way to the malignant transformation of the cell [9].One of the genes whose promoter in tumor cells is frequently methylated is MGMT. Studies have shown that due to the MGMT gene promoter methylation, the reduction of its expression occurs in approximately 45% of patients with GBM, which results in the absence of repair of the damage caused by TMZ. Therefore, the introduction of this drug prolongs the lives of these patients, while in the remaining 55%, due to the high levels of MGMT activity, there is no therapeutic effect [10]. However, even determination of the methylation status of the MGMT promoter does not guarantee that those patients in which the treatment with TMZ will have positive effects will be selected with certainty, given that recent studies have shown that there is not always a correlation between the methylation status of the MGMT and the expression of the protein which that gene encodes [11].

Therefore, a large number of studies that deal with this issue aim at overcoming the problems which, in the treatment of with TMZ and other alkylating agents, cause the activity of MGMT [12, 13].

Unfortunately, for the time being, there is no indication that the results of these studies will lead to any significant breakthroughs in the treatment of GBM, and the modest results in prolonging survival after the introduction of TMZ to the treatment of this tumor are far from the expectations of the patients and their families.

There are more and more reports showing the use of herbal remedies in the treatment of various tumors. The majority of them indicates the potentials found in the plants of the genus *Artemisia* L. [14, 15]. These plants achieve their antitumor activity through its active metabolite dihydroartemisinin (DHA) by inhibiting tumor cell proliferation [16]. The fact that DHA is able to pass through the blood-brain barrier and achieve its efficiency in brain tumors is especially important [17]. It is worth mentioning that artemisinin and its derivatives enhance the glioblastoma cells sensitivity to radiotherapy [18]. There are some reports implying that DHA increases TMZ effects on glioma cells in rats, but there is not enough data on human brain tumors efficiency by DHA alone or combined with TMZ [19].

The aim of the research is to demonstrate the possibility of stopping the tumor progression and decreasing tumor mass with the help of pharmacologically active ingredients found in appropriate herbal remedies.

## Methods

The research was conducted in the period from 2010 to the end of 2016. Prior to the start of phytotherapy (PT), all the patients submitted their medical records with a diagnosis made on the basis of the inspection of a sample of tumor tissue and a scan of the affected area using nuclear magnetic resonance (NMR) and/or computed tomography (CT). These data were used as the basis for a comparative monitoring of the effectiveness of PT in terms of comparing the dimensions of the tumor prior to PT with the results of the control scans that patients underwent during and after PT.

The preparation of herbal medicine is carried out in several stages, starting with the selection of the best plants as the ingredients. In the selection of the plants, priority was always given to those that can be found in nature, i.e., plants that are wildcrafted. In comparison with other plant species, such plants obtained the resources necessary for their growth and development on their own, by which passed the natural selection process and became the finest representatives of their sort. Moreover, the plants grown in nature are harvested manually. Trained harvesters make a selection at the spot by harvesting fully mature plants only, avoiding sick or damaged ones. Cultivated plants are usually harvested by machines. Thereby, all the plants are harvested, and the separation of quality plants from non-quality ones is completed later. Additional selection and cleaning of these plants do not guarantee to obtain quality raw material because at this point, it will be hard to recognize less quality plants.

Approximately 80% of plants that are part of herbal medicine are wild sorts, while the remaining 20% are obtained by breeding.

The next stage involves drying the plants. All of the plants are dried in a natural way, without any additional energy sources. The drying process is the most common type of medicinal plant conservation. The humidity of the plants at the moment of harvesting is about 60–80%; after the drying process, it should not be over 10% because this humidity ensures plant conservation for longer periods of time. Wrongly dried plants will rot easily, and they lose medicinal properties. The plants should be dried so that they can keep active materials and color. Folium, flos, and herba are dried in a thin layer alongside intensified air currents. During the drying process, these parts of the plant should not be exposed to direct sunlight. While drying aromatic plants, we take account of temperatures not exceeding 40 Celsius degrees, otherwise the essential oils would evaporate and disappear.

Before bringing the dried plants into the storage room, they are sterilized by rapid cooling to − 15 °C. From the raw material prepared in such a manner, preparations are made just before their application. All the plants that are part of herbal medicine, whether they are wild sorts or obtained by breeding, are from Bosnia and Herzegovina.

### Standard phytotherapy

The patients were treated with two different combinations of herbal medicines. The first combination was marked as standard phytotherapy (StPT). This combination of herbal medicines consisted of five types of herbal mixtures that differed in composition (preparation 1, preparation 2, preparation 3, preparation 4, and preparation 5).

The herbal remedy ingredients are given in Tables 1, 2, 3, 4, and 5.

**Table 1:** Ingredients of preparation 1

Preparation 1				
Pharmaceutical name	Botanical name	Family	Part used	Percentage representation, (%)
Herba artemisiae-alba	*Artemisia absinthium* L.	Asteraceae	Herba	25
Herba Artemisiae vulgaris	*Artemisia vulgaris* L.	Asteraceae	Herba	25
Visci albi herba	*Viscum album* L.	Santalaceae	Herba	25
Centaurii herba	*Erythrea centaurium* L.	Gentianaceae	Herba	25

**Table 2:** Ingredients of preparation 2

Preparation 2				
Pharmaceutical name	Botanical name	Family	Part used	Percentage representation, (%)
Herba catariae	*Nepeta cataria* L.	Lamiaceae	Herba	20
Melissae folium	*Melissa officinalis* L*.*	Lamiaceae	Folium	15
Thymi herba	*Thymus vulgaris* L.	Lamiaceae	Herba	10
Origani herba	*Origanum vulgare* L.	Lamiaceae	Herba	10
Matricariae flos	*Matricaria chamomilla* L.	Asteraceae	Flos	10
Lupuli strobili	*Humulus lupulus* L.	Cannabaceae	Storobili	10
Rosmarini folium	*Rosmarinus officinalis* L.	Lamiaceae	Folium	5
Calendulae flos	*Calendula officinalis* L.	Asteraceae	Flos	5
Valerianae radix et rhzoma	*Valeriana officinalis* L.	Valerianaceae	Radix et Rhizoma	5
Bursae pastoris herba	*Capsella bursa pastoris* L.	Brassicaceae	Herba	5
Basilici herba	*Ocimmum basillicum* L.	Lamiaceae	Herba	5

**Table 3:** Ingredients of preparation 3

Preparation 3				
Pharmaceutical name	Botanical name	Family	Part used	Percentage representation, (%)
Althaeae radix	*Althaea officinalis* L.	Malvaceae	Radix	15
Althaeae folium	*Althaea officinalis* L.	Malvaceae	Folium	15
Betulae folium	*Betula pendula* Roth	Betulaceae	Folium	15
Hyperici herba	*Hypericum perforatum* L.	Hypericaceae	Herba	15
Menhtae piperitae folium	*Menhta piperita* L.	Lamiaceae	Folium	15
Herba glechomae	*Glechoma hederacea* L.	Labiatae	Herba	15
Cheliodonii herba	*Chelidonium majus* L.	Papaveraceae	Herba	10

**Table 4:** Ingredients of preparation 4

Preparation 4				
Pharmaceutical name	Botanical name	Family	Part used	Percentage representation, (%)
Urticae herba	*Urtica dioica* L.	Urticaceae	Herba	20
Millefolii herba	*Achilea millefolium* L.	Compositae	Herba	20
Betulae folium	*Betula pendula* Roth	Betulaceae	Folium	30
Teucrii montani herba	*Teucrium montanum* L.	Lamiaceae	Herba	15
Centaurii herba	*Erythrea centaurium* L.	Gentianaceae	Herba	15

**Table 5:** Ingredients of preparation 5

Preparation 5				
Pharmaceutical name	Botanical name	Family	Part used	Percentage representation^a^, (%)
Herba catariae	*Nepeta cataria* L.	Lamiaceae	Herba	25
Melissae folium	*Melissa officinalis* L*.*	Lamiaceae	Folium	20
Thymi herba	*Thymus vulgaris* L.	Lamiaceae	Herba	15
Matricariae flos	*Matricaria chamomilla* L.	Asteraceae	Flos	15
Lupuli strobili	*Humulus lupulus* L.	Cannabaceae	Storobili	10
Rosmarini folium	*Rosmarinus officinalis* L.	Lamiaceae	Folium	5
Calendulae flos	*Calendula officinalis* L.	Asteraceae	Flos	5
Bursae pastoris herba	*Capsella bursa pastoris* L.	Brassicaceae	Herba	5

^a^Weighted percentage

The patients prepared all five herbal medicines and took them in the form of tea every day at regular intervals. The patients took preparation no. 1 at 7 a.m., no. 2 at 10 a.m., no. 3 at 1 p.m., no. 4 at 4 p.m., and no. 5 at 7 p.m. (Table 6). In patients who experienced a progression of the disease, the treatment was continued with a combination of selected herbal medicines marked as phytotherapy of salvation (PTS). This group of herbal medicines consisted of the first four preparations that are included in the composition of standard phytotherapy, while the fifth preparation was not included. The patients took this combination of herbal medicines five times a day, as well, and in the following manner: they took preparation no. 1 two times a day at 7 a.m. and 7 p.m., and preparation nos. 2, 3, and 4 once a day at 10 a.m., 1 p.m., and 4 p.m., respectively. Hereby, the daily dose of preparation no. 1 was doubled (Table 6).

**Table 6:** Time of herbal remedy consumption and dosage

	Standard phytotherapy (StPT)				
Herbal medicine no.	1	2	3	4	5
Time of taking tea	Every day at 7 a.m.	Every day at 10 a.m.	Every day at 1 p.m.	Every day at 4 p.m.	Every day at 7 p.m.
Daily dose of tea (cm^3^)	200	200	200	200	200
	Phytotherapy of salvation (PTS)				
Herbal medicine no.	1	2	3	4	1
Time of taking tea	Every day at 7 a.m.	Every day at 10 a.m.	Every day at 1 p.m.	Every day at 4 p.m.	Every day at 7 p.m.
Daily dose of tea (cm^3^)	200	200	200	200	200

The preparations consist exclusively of crushed parts of the plants, without any other additives. The plants included in the composition are pounded to a standard degree. Sieve no. 6 (rough cut) was used for flowers, stems, and leaves; sieve no. 3 was used for roots and bark; and sieve no. 2 (fine cut) was used for seeds and fruits [20]. All preparations are prepared in the same manner, and to prepare a single dose of tea, 1.5 g of herbal mixture and 200 cm^3^ of water is needed. The basis of StPT and PTS are herbal mixtures (preparations 2, 3, and 5) which yielded good results in the treatment of macroprolactinoma [21].

During the follow-up, i.e., when comparing the condition of patients before and after PT, the following key indicators were used:

- Information on preoperative tumor size;
- Information on the extent of cerebral edema, including usage and dose of corticosteroids;
- Information on previous and current oncological treatment;
- Information on the duration and side effects of PT;
- Information on values of liver markers;

In addition to these indicators, records on some other indicators were kept (e.g., beginning and duration of PT, age and sex of patients, tumor progression).

### Statistical analysis

Since five cases are presented in this study, there was no foundation for statistical analysis construction.

## Results

The results of the research are shown in the “Case presentation” section.

### Case presentation

#### Case 1

**Fig. 1:**
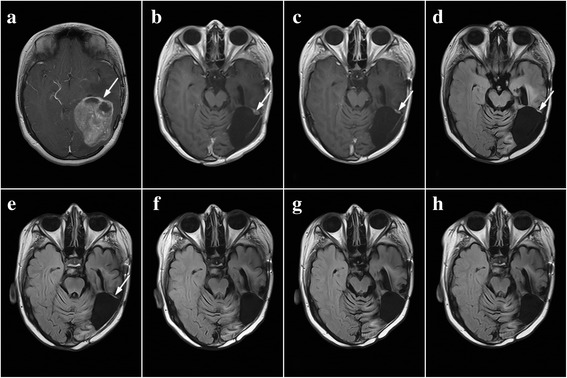
Chronological summary of NMRI findings of patient no. 1. Tumor tissue is indicated by arrows. **a** March 2010, scan of tumor pre-surgery. **b** September 2010, the presence of tumor residue was found, dimensions 8 × 6 mm. **c** December 2010, the tumor was approximately the same dimensions as on the previous scan. **d** May 2011, a reduction of the tumor to 8 × 4 mm was determined. **e** September 2011, a further reduction of the tumor to the dimensions 7 × 2 mm. **f** September 2012, b no radiological signs of a tumor. **g** February 2013, 3 years after the diagnosis, there are no signs of tumor. **h** March 2014, 4 years after the diagnosis, there are no radiological and clinical signs of tumor

The first report describes the case of a 15-year-old girl who developed glioblastoma multiforme (GBM) from the previously treated diffuse astrocytoma (Gr-2). The patient underwent the first surgery at the Neurosurgery Clinic in November 2005 due to the diagnosed diffuse astrocytoma. The second surgery was performed in August 2008, and an inspection of a sample of tumor tissue showed that it was a recurrence of a diffuse astrocytoma. Afterwards, the girl underwent regular checkups and was evaluated on the basis of the findings of MRI that was performed on several occasions after the last surgery. In the early 2010, the patient’s condition worsened. She had a few epileptic seizures, and she was showing signs of sleepiness and had trouble concentrating. On 26 March 2010, an urgent MRI was performed, and it showed a massive recurrence of glial tumor measuring 70 ×60 × 50 mm (Fig. 1a).

Meanwhile, there was a sudden deterioration of consciousness of the patient which progressed to the stage of coma caused by spontaneous bleeding from a pathological process in the brain, which is why on the patient underwent an emergency surgery 01 April 2010, during which a left-side decompression parietal-temporal-occipital craniotomy, evacuation of hematoma, and the reduction of an expansive process were performed. As the postoperative CT scan of the brain showed the persistence of cerebral edema and the expansive process, a decompression re-craniotomy and an additional resection of the expansive process were performed. After this procedure, the second control postoperative CT was performed, and since the findings were satisfactory, the girl was awakened and taken off the controlled mechanical ventilation.

On 14 April 2010, the patient underwent a new surgical procedure in terms of the maximum reduction of the tumor mass. The samples of tumor tissue were sent for intraoperative analysis, and later to detailed histopathological analysis. After the analysis of the samples obtained, it was concluded that it was a highly anaplastic glial tumor with an expressed vascular proliferation and larger areas of focal hemorrhagic necrosis. A very high mitotic activity was observed within the tumor. In the tumor portion along the necrosis margins, an abundance of foam cells and hemosiderophages, as well as gliosis, was found. From the aspect of immunocytochemistry, 20% of tumor cells gave a positive response to p53. The final diagnosis was glioblastoma multiforme (GBM).

In late April 2010, the patient started to use herbal medicine. The StPT combination was introduced, and she used it in the course of following 33 months.

Between 17 May 2010 and 23 June 2010, the girl was hospitalized at the radiology ward, where 3D conformal radiotherapy of the brain tumor (glioblastoma multiforme) in the left parietal-temporal-occipital region of the brain was performed by using a linear accelerator with a power of 6 MW. A therapeutic dose of 56 Gy was administered in 28 fractions, along with the accompanying therapy with temozolomid (TMZ) capsules of 75 mg/m^2^ of body surface area. During therapy, nausea and occasional vomiting occurred once a day, which was put under control by antiemetics. She was discharged in a good general condition. In addition to radiation therapy and the therapy with TMZ, the patient also used herbal medicine. After completion of the combined radio and chemotherapy (RT/CT), the patient underwent no further oncological treatment but continued with PT. As a medical treatment, she used antiepileptic drugs and a dexamethasone dose of 4 mg/day. In September 2010, a control scan was performed which showed the presence of tumor residues, dimensions 8 × 6 mm (Fig. 1b). In this period, a significant reduction of brain edema volume occurred; furthermore, alongside PT, the patient kept using antiepileptic drugs only. During this period, there had been a significant reduction of the volume of cerebral edema, so the patient, in addition to PT, continued taking only antiepileptic drugs. The control nuclear magnetic resonance imaging (NMRI) performed on 23 December 2010 (Fig. 1c) showed that the tumor was approximately the same dimensions as on the previous scans. The following control scans showed continuous tumor regression. The NMRI performed on 17 May 2011 (Fig. 1d) showed that the tumor dimensions were 8 × 4 mm, and the scan performed on 20 September 2011 (Fig. 1e) showed that the tumor dimensions were 7 × 2 mm. The disappearance of the tumor was determined by a control scan on 19 September 2012 (Fig. 1f), and NMRIs performed on 25 February 2013 (Fig. 1g) and 11 March 2014 (Fig. 1h) showed no radiological signs of a tumor. The patient used phytotherapy for 27 months with full capacity and without breaks, as long as radiological signs of the tumor were present. After this, she kept using all five herbal remedies for 6 months, but every other day, after this, the PT was concluded.

#### Case 2

**Fig. 2:**
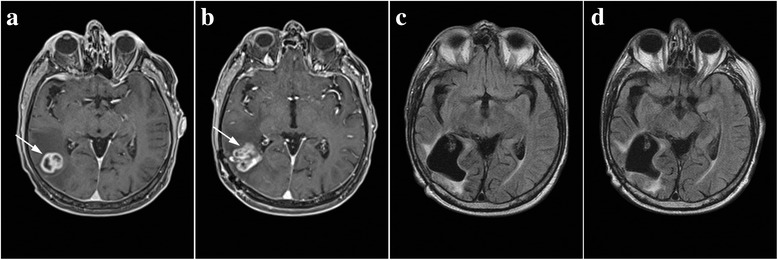
Chronological summary of NMRI findings of patient 2. Tumor tissue is indicated by arrows. **a** February 2012, scan of tumor pre-surgery. **b** June 2012, scan after GBM recurrence. **c**, **d** Control scans performed 3 and 4 years after the initial diagnosis show no radiological signs of tumor

The NMRI performed on a man aged 63 years due to frequent headaches and troubles with vision in February 2012 established the presence of a tumor measuring 26 × 24 mm (Fig. 2a). The tumor was located in the temporal-occipital region on the right side. The patient underwent surgery on 21 February 2012, and on that occasion, a complete resection of the tumor was performed. Histopathological analysis showed that it was glioblastoma multiforme (WHO grade IV). After 4 weeks, the treatment was continued with combined RT/CT, along with a daily dose of TMZ of 75 mg/m^2^ of body surface area. As, during the course of treatment, the patient experienced pronounced side effects (thrombocytopenia); after the completion of RT/CT, the planned monotherapy with TMZ was not conducted. On 20 June 2012, due to the recurrence (Fig. 2b), the patient underwent a new surgery, which marked the end of the oncological treatment. During another operation, the visible part of the tumor has been removed entirely (complete resection). The patient started to use phytotherapy immediately after the second surgery. A combination of herbal medicines marked as standard phytotherapy was introduced, which he took every day without interruption for the following 24 months. After this, he kept taking all five herbal remedies for 6 months, but every other day, after this, the PT was concluded. We should mention that the patient was using dexamethasone with a dose of 4 mg/day at the beginning of phytotherapy. Six months later, dexamethasone was excluded and he did not use it anymore until the end of phytotherapy. The patient periodically underwent control NMRI, which showed that, even 48 months after the initial diagnosis and the surgery, there were no signs of recurrence (Fig. 2c, d).

#### Case 3

**Fig. 3:**
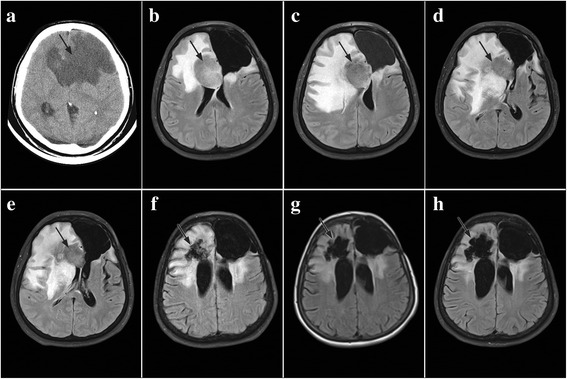
Overview of control scans of patient 4. Tumor tissue is indicated by arrows. **a** CT performed in March 2012, scan of tumor pre-surgery. **b** The NMRI from August 2012 shows the progression of the tumor, after which StPT was introduced. **c** The NMRI performed in November 2012 shows stopping of the progression after the introduction of StPT. **d** The NMRI from February 2013 shows a new progression of the tumor after which PTS was introduced. **e** The NMRI scans performed in May 2013 show regression of the tumor to 50 mm. **f** The control NMRI performed in October 2014 showed the further reduction of the tumor to 45 mm. **g** The NMRI performed in April 2015 showed that the tumor was approximately of the same dimensions as on the previous scan. **h** The scans performed in May 2016, 4 years after the initial diagnosis, showed that the tumor radius remained approximately of the same dimensions as on the previous two scans

After problems that manifested with headaches, difficulty walking, weakness of the right limbs, and mental disorders in a woman aged 46 years, on 15 March 2012, a head scan was performed by the method of computed tomography (CT). On that occasion, an extensive expansive lesion bilaterally and frontally was observed, predominantly on the left side with propagation to the left temporal and left parietal lobe, measuring 90 × 80 mm (Fig. 3a). On 27 March 2012, a maximum tumor resection was performed. Extempore analysis of tumor tissue established that it was glioblastoma [*bihemispheric glioblastoma* (*butterfly glioma*)], and the opinion of a pathologist given a few days after the surgery suggested that it was a diffuse astrocytoma (gr II). Following the surgery, the patient was hospitalized in the radiology ward, where 3D conformal radiotherapy was performed by using a linear accelerator with a power of 6 MW. A therapeutic dose of 46 Gy was administered in 23 fractions, followed by radiation of the tumor base with a therapeutic dose of 8 Gy in 4 fractions. With this, the oncological treatment was completed. In August 2012, the patient’s condition deteriorated. At an emergency MRI imaging on 23 August 2012, the progression of the tumor was established, and the dimensions were 46 × 36 mm (Fig. 3b). After her physician informed her about the options of tumor treatment at this stage, the patient refused further oncological treatment and applied for phytotherapy (PT) on 26 August 2012.

StPT was introduced, and she used it in the course of following 6 months. The control MRI imaging performed 3 months after the introduction of StPT (on 21 November 2012) found that tumor progression had stopped (Fig. 3c); therefore, the patient continued to use the combination of herbal medicines. In February 2013, the patient’s condition began to deteriorate, and treatment with corticosteroids was urgently introduced. A daily dose of dexamethasone of 8 mg/day was introduced, and it was quickly raised to 16 mg/day. MRI imaging of 22 February 2013 showed the progression of the tumor, whose radius was 60 mm and which was surrounded by a large perifocal edema that was completely compressing the right lateral cerebral ventricle (Fig. 3d). Following these findings, the composition of the preparation was modified, and a PTS combination of herbal medicines was introduced instead of StPT. Shortly, after introducing PTS and increasing dexamethasone doses, the patient felt an improvement that mainly manifested by a higher mobility of the limbs. The control NMRI from 21 May 2013 (Fig. 3e) showed a reduction of the tumor from 60 to 50 mm, and the scan from 01 October 2014 showed that the tumor radius was 45 mm (Fig. 3f). Further control scans performed during 2015 and 2016 (Fig. 3g, h) showed that the tumor radius was approximately of the same dimensions as on the scan from October 2014. Along tumor regression, decreasing of brain edema followed, so the dexamethasone dose was gradually decreased, and it was completely excluded at the end. The patient used PT with full capacity and without breaks for 48 months. After some control scanning showed that tumor dimensions were not changed, a reduced dose of teas (all of five teas, but every other day) was introduced, which she also used at the time of delivering this report.

#### Case 4

**Fig. 4:**
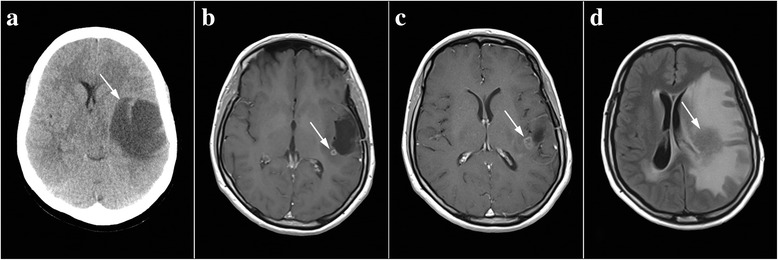
Overview of control scans up to the introduction of PT. Tumor tissue is indicated by arrows. **a** CT scan of a diffuse astrocytoma. **b** The NMRI after the completion of oncological treatment indicated a recurrence measuring 5 mm. **c** Recurrence 11 mm. **d** Progression of the tumor

**Fig. 5:**
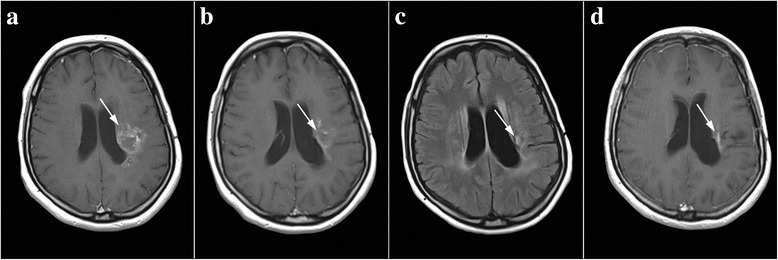
Overview of control NMRI scans after the introduction of PT. Tumor tissue is indicated by arrows. **a** Control scan after the completion of a combined therapy with TMZ and FT and regression of the tumor. **b**, **c**, and **d** Continuance of the regression until the complete absence of clinical and radiological signs of the tumor had been achieved solely with PT.

Due to frequent headaches, epileptic seizures, speech disorders, and stiffness of the right half of the body in a 33-year-old woman, emergency imaging by a computed tomography (CT) was performed in June 2011. The scans showed a well-limited tumor mass without necrosis, located to the left in the frontal parietal-temporal region, measuring 56 × 45 × 51 mm (Fig. 4a). A month later, the patient underwent surgery during which a complete resection of the tumor was performed. After the inspection of a sample of tumor tissue, a diffuse astrocytoma Gr-II was diagnosed. After surgery, the patient was hospitalized in the radiology ward, where 3D conformal radiotherapy was performed using a 6 MW linear accelerator. The oncological treatment was completed by administering a therapeutic dose of 54 Gy in 27 fractions. A control scan using the nuclear magnetic resonance imaging (NMRI) technique performed in April 2012 (Fig. 4b) showed the presence of local recurrence, with a diameter of 5 mm, and the scan from September 2012 showed an increase of the tumor to 11 mm (Fig. 4c). In February 2013, due to the weakness of the right limbs and speech disturbances, an emergency NMRI was performed and found the presence of an extensive cerebral edema and recurrent tumor, 40 mm in diameter, which was spreading towards the basal ganglia, and the midsagittal plane shifted approximately 9 mm to the right (Fig. 4d). The patient immediately started receiving antiedema therapy and antiepileptics (dexamethasone 16 mg/day), and oncology treatment was continued with the introduction of TMZ in 6 cycles of 28 days at a dose of 200 mg/m^2^ of body surface area, for 5 days during each cycle. In March 2013, the patient applied for PT and began taking it along with TMZ. A combination of herbal medicines marked as phytotherapy of salvation was immediately introduced. A control NMRI performed in October 2013 showed that there had been a regression of the tumor, with the diameter being 22 mm at the time (Fig. 5a). The dexamethasone dose was decreased to 8 mg/day. After 6 cycles of therapy with TMZ, the patient completed oncological treatment in September 2013, and further treatment consisted solely of PT. The only pharmaceutical drugs she continued to take were antiepileptic drugs. Subsequent control scans performed in February and October 2014 showed continuous tumor regression (Fig. 5b, c). Finally, 30 months after the introduction of PT, the tumor could not be detected on the control scan from 31 August 2015, and the irregular dotted area that postcontrastly raised the signal intensity was recognized by a physician as a scar from the previous surgery from 2011 (Fig. 5d). This patient used herbal remedies with full capacity until there were no radiologically signs of a tumor, respectively 30 months. After this, she kept taking all of the five teas for 6 months, but every other day, which concluded the PT.

#### Case 5

**Fig. 6:**
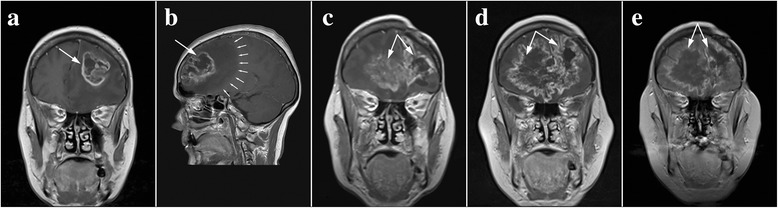
Chronological summary of the NMRI for patient 5. Tumor tissue is indicated by arrows. **a**, **b** August 2011, a scan of tumor pre-surgery and introduction of StPT immediately after the surgery. **c** April 2014, a scan of the bilateral and frontal recurrence and re-introduction of StPT. **d** November 2015, the progression of the tumor and introduction of PTS. **e** April 2015, stopping the progression of recurrence

Due to persistent headaches and the qualitative alteration of consciousness in a woman aged 58 years, on 11 July 2011, a head NMRI was performed, and it showed an expansive lesion in the left frontal portion of the brain, measuring 60 × 50 × 40 mm (Fig. 6a, b). The tumor was accompanied by a large edema (Fig. 6b). As the first step, dexamethasone was included with a dose of 8 mg/day. The surgical procedure was performed on 25 August 2011, and a maximum tumor resection was performed on that occasion.

In the period between 08 November and 23 December 2011, the patient underwent radiation with 46 Gy, followed by radiation of the tumor base with 14 Gy in 14 fractions. During radiation, the patient had been receiving TMZ in a dose of 120 mg daily for 42 days. Afterwards, 6 cycles of TMZ (240 mg during each cycle) were administered.

The patient started to use standard phytotherapy together with combined RT/CT, and then together with monotherapy with TMZ, and continued to use it after the completion of the oncological treatment. She had been taking PT at full capacity and without interruption for 24 months. Five months after the completion of PT, the patient began to complain of an intense headache; so in April 2014, a control NMRI was performed, and a recurrence of the underlying disease in the frontal portion of the head on both sides, in the shape of a butterfly, was found on that occasion. On the right side, prominent was a larger area of 56 × 47 × 43 mm in diameter that occupied the front third of the corpus callosum (Fig. 6c).

After these findings, the patient was reintroduced to oncological treatment, which consisted of the combined RT/CT followed by planned 6 cycles of monotherapy with TZM in a daily dose of 260 mg for 5 days during a cycle of 28 days. After the third cycle of the treatment with TZM, a control MRI was performed on 04 November 2014, which showed that the dimension of the larger area, located in the right frontal lobe, which occupied the area of the corpus callosum, was 73 × 49 mm (Fig. 6d). Due to the progression of the tumor, the treatment with TMZ was not continued. With this, the oncological treatment was completed, and medical treatment continued with the regular intake of antiepileptic drugs and, when necessary, antiedema therapy with synthetic corticosteroids.

The patient continued to use standard phytotherapy immediately after the recurrence had been diagnosed. After control imaging performed on 04 November 2014 that showed there had been a progression of the tumor, a PTS combination of herbal medicines was introduced instead of StPT.

Control MRI imaging from 26 March 2015 showed that further progression of the tumor had stopped. We should mention that the blocking of tumor growth occurred at the time when PT was the only way of treatment (Fig. 6d). In the course of the next 4 months, the patient was relatively stable, and the occasional crises were repressed by introducing, or increasing, the dose of corticosteroids (at first 8, and 16 mg/day of dexamethasone afterwards). However, in early August 2015, there was a sudden deterioration, the patient fell into a coma and died in mid-August 2015.

## Discussion

While in the case of diffuse astrocytoma, after malignant transformation, the standard treatment protocol which is used in primary high-grade astrocytoma is administered; with the progression of primary glioblastoma (GBM), there is no clear strategy of treatment, so the decision on the choice of therapy has to be made by a physician on the basis of the general condition of the patient, the location of the tumor, and previous treatment. As a second-line therapy (so-called rescue therapy), chemotherapy with temozolomide (TMZ) had been commonly used, and it is the only effective drug in the treatment of recurrent glioblastoma [22]. The reports on the achieved results of the treatment of GBM with TMZ after the recurrence of the disease show temporarily cessation of tumor growth in a number of patients. The introduction of a continuous intensive TMZ dose of 50 mg/m^2^ per day as a “rescue” treatment should be noted as one of the most valuable results, which stop further progression for a period of 6 months in 36% of patients [23]. In patients in whom, in the course of treatment with TMZ, there has been a progression of GBM, as well as in those who experienced pronounced side effects during the first chemotherapy treatment, another surgery is performed as a rescue therapy.

This paper reveals the use of phytotherapy (PT) in the treatment of five patients suffering from brain tumors who, in addition to varying according to the formation of tumors and the method of treatment, also varied according to the degree of cerebral edema which, how it turned out in the course of this research, significantly affected the effectiveness of PT and was a significant factor that helped to determine the composition of herbal medicines for individual patients. In the course of the research, care was taken not to deprive subjects of the best method of treatment currently used in the treatment of brain tumors in modern medicine. Thus, patients 1, 4, and 5 used PT together with oncological treatment and continued to use it after the treatment had ended, while those patients who completed the oncological treatment (patients 2 and 3) were treated solely with PT. There are some information claiming that pharmacologically active ingredients of the *Hypericum perforatum* L. plant can decrease the antitumor drugs and antiepileptic activity [24]. This plant is included in the number 3 remedy. Because of this reason, the patients exposed to chemotherapy and those who took antiepileptic drugs were given the number 3 remedy without *Hypericum perforatum* L. This was the only change in the composition of herbal remedies.

The first two patients whose cases are described in this research paper were continuously treated with a combination of herbal medicines marked as StPT. Among the patients suffering from GBM and high-grade astrocytic tumors in general, there is a difference in the length of survival, and to date, several prognostic factors affecting the survival rate have been identified [25]. Identifying prognostic factors and estimating the length of survival based on these factors are important for the evaluation of the effectiveness of certain treatment methods and the introduction of new drugs in the treatment of GBM. In the last few years, it has been used a data of mutation of the genes that code isocitrate dehydrogenase 1 and 2 (IDH 1 and IDH 2) as an important prognostic factor for GBM. Researches have shown that patients with IDH mutation gliomas live longer than patients having an IDH wild-type of glioma [26]. A valuation of mutation of the gene that codes IDH 1 and IDH 2 has been introduced in clinical praxis after our patients have been diagnosed; furthermore, we could not get these genes status data.

Several studies point to pre-surgery tumor size as a significant independent prognostic factor in assessing the length of survival. The trials performed on 510 subjects with malignant glioma, 80% of whom had a diagnosis of GBM, showed that tumor size is a significant prognostic indicator, independent of other prognostic variables [27].

The significance of pre-surgery tumor size with respect to the length of survival was also pointed out in a trial performed on 76 subjects suffering from high-grade astrocytoma, 51 of whom were diagnosed with GBM [28].

A mathematical model that took into account the rate of tumor growth and diffusion coefficient predicted that the length of survival of patients with GBM is 158 days on average, which corresponds to the results of the aforementioned trials [29]. The mean tumor diameter in the female subject whose case was first described in this paper was 60 mm (D_1-3max_ = (a + b + c)/3), which placed her in the group of patients with a poor prognosis and shorter survival expectancy.

The extent of cerebral edema at the time of diagnosis represents a significant prognostic factor influencing the time interval of recurrence and the length of survival of patients suffering from GBM, because it suppresses tumor infiltration zone and creates conditions for the migration of tumor cells to portions of the brain that are beyond the reach of surgical procedure and radiotherapy. Therefore, it is considered that the extent of cerebral edema is directly proportional to the presence of tumor cell infiltrates. It should be noted that, due to a shorter period between surgery and recurrence, the patients whose volume of cerebral edema exceeds 75 cm^3^ survive for a significantly shorter period [30].

In the first patient, an extensive edema was found by NMRI, and it affected the left hemisphere of the brain almost in its entirety, so that a rapid recurrence had been realistically expected, although it did not happen even after 48 months from the initial diagnosis.

Given the pre-surgery tumor size, larger areas of hemorrhagic necrosis, an edema of nearly the entire left hemisphere of the brain, and incomplete oncological treatment, because combined RT/CT had not been followed by monotherapy with TMZ, as well as the poor general condition of the patient, we have a reason to believe that the regression of the tumor residue and long survival of the first subject without the progression of the disease occurred due to the benefits of phytotherapy.

Oncological treatment was also not completed in the second patient. It is possible that this was the reason for the recurrence of GBM 4 months after the surgery. After the recurrence, another surgery had been chosen as the second line of defense given that, during the combined RT/CT, the patient had trouble tolerating TMZ, which is the most common choice of treatment in GBM recurrence. Most studies that have dealt with the analysis of the efficacy of a second surgery in the treatment of GBM, including a major trial performed on 208 patients, point out that it does not extend the survival of patients, i.e., that the benefits of a second surgery with respect to GBM for patients are minimal or non-existent [31]. Given that the patient, despite the return of the tumor shortly after the surgery, showed no signs of the progression of the disease 48 months after the initial diagnosis, it is quite safe to say that the use of herbal medicines contributed to such a long survival.

### Patient treated with phytotherapy of salvation

In the third subject, the progression of tumor occurred soon after RT was completed (Fig. 3b), so a new surgical procedure had been proposed to the patient. After a physician informed the patient about the risks another surgery posed, the patient refused further treatment and commenced with PT in August 2012. StPT was introduced as the first choice of treatment. This was the only subject out of five who, after the progression of the tumor, did not continue with oncological treatment. For the whole duration of PT, the only pharmaceutical drugs the patient had been taking were antiepileptic drugs and, when necessary, corticosteroids.

In this subject, there had been different assessments of tumor grade. A surgical report clearly indicated a highly vascularized tumor, necrotic in its central portion, which suggested it was most likely GBM, and which was confirmed by the extempore analysis. On the other hand, the pathologist claimed that the obtained sample showed no necrosis, that the proliferation of blood vessels was not expressed, and that the proliferative index, measured by Ki-67 antibodies, was low, which led him to the conclusion that it was a diffuse astrocytoma (gr-II).

It is known that, inside a tumor, there are often regions with different extent of differentiation and that, in such cases, tumor grade is determined by the least differentiated region. In his report, the surgeon clearly mentioned the presence of necrosis, which is incompatible with the diagnosis of diffuse and anaplastic astrocytoma [32]. In the pathology report, the pathologist claimed that there were no signs of necrosis in the tissue sample he had received, which suggests that the procedure of taking tissue from the portion of the least differentiated region was not observed, which led the pathologist to make the wrong conclusion with respect to the grade of the tumor.

The progression of tumor shortly after the completion of radiotherapy is an additional argument that, in the case of this patient, the tumor was GBM. Despite an encouraging cessation of tumor growth after the introduction of StPT (Fig. 3c), 6 months later, the patient experiences a further increase of the volume of cerebral edema, which was highly compressive and was completely compressing the right lateral cerebral ventricle (Fig. 3d). Cerebral edema significantly reduces the concentration of drugs used by modern medicine to fight tumors of the brain by putting the pressure on the blood vessels, which leads to the creation of hypoxic zones and, consequently, to a reduced flow of drugs to the tumor cells [33]. In this specific case, it is likely that cerebral edema obstructed the flow of pharmacologically active ingredients from herbal medicines, which reduced their effectiveness. In order to overcome this problem, it was decided that the composition of the mixture should be modified and a phytotherapy of salvation (PTS) should be introduced instead of StPT. This modification doubled the daily dose of preparation 1, which was believed to have the greatest potential in the fight against the tumor, but the daily amount of tea remained the same. At the same time, the patient’s dose of dexamethasone was raised to 16 mg/day. Shortly after this intervention, the general condition of the patient began to improve. Quite surely, the new combination of herbal remedies is responsible for this improvement in health, but also for the reduction of brain edema volume that occurred after introducing high doses of dexamethasone. The efficacy of the new combinations of herbal medicines was confirmed by a subsequent control NMRI which was performed 3 months later, which showed tumor regression (Fig. 3e). In the following 18 months, the tumor radius had reduced for another 5 mm (Fig. 3f), and control scans performed during 2015 and at the beginning of 2016 (Fig. 3g, h) showed that the tumor dimensions did not change. At the time of submission of this paper, the patient had still been using herbal medicines, 4 years since the introduction of PT.

The results achieved with this subject are the most significant part of this paper, not only because of the cessation of tumor progression and its reduction, which occurred exclusively owing to the use of PT, but also because this subsequently introduced combination of herbal mixtures has become a model for the treatment of patients whose tumor is accompanied by a high degree of cerebral edema and those who have an extremely poor prognosis.

The fourth patient applied for PT after the malignant transformation of previously treated diffuse astrocytoma (Fig. 4a). The control NMRI performed in February 2013 (Fig. 4d), compared to the scan from September 2012 (Fig. 4c), showed a significant increase in tumor size accompanied by extensive edema which affected most of the left hemisphere of the brain. A progression to a higher grade had occurred quite quickly, which was expected, given that in diffuse astrocytoma, an average proliferative index measured by Ki-67 antibodies is around 2.5%, while in this patient, the number of divided cells was 4–5%, suggesting the possibility of a faster malignant transformation [32]. After the progression, the patient was introduced to TMZ in 6 cycles, 28 days each, with 200 mg/m^2^ of TMZ a day for 5 days of each cycle.

PT was introduced together with TMZ. Since the previously described cases showed that StPT has a limited effect on tumors accompanied by a large brain edema, the first choice of treatment for this patient was PTS. The patient started using herbal medicines together with TMZ, which leads to the conclusion that the tumor regression during that time is due to the combined usage of PT and TMZ (Fig. 5a). The description of the subject’s case is, according to our discovery, the first evidence of successful synergy of herbal remedies and chemotherapy in human brain tumors.

However, the further reduction of the tumor during the period in which the only method of treatment was PT can be attributed exclusively to herbal medicines (Fig. 5b–d).

In the fifth patient, due to the pre-surgery size of the tumor and a large perifocal edema (Fig. 6a, b), a rapid progression was expected since these prognostic factors had put her in a group of patients with poor prognosis, whose survival was 6 months on average, regardless of the administered treatment. Contrary to expectations, a recurrence was diagnosed 30 months from the initial diagnosis. It is certain that the use of herbal remedies, combined activity of radio/chemotherapy and PT, as well as the continuous usage of herbal remedies after the end of oncological therapy, contributed to such a long period without recurrence. Control scans performed in April 2014 showed the presence of a massive tumor that affected both hemispheres of the brain (Fig. 6c).

Since there were indications that the previous phytotherapy previously had helped the patient, StPT was reintroduced immediately with a combined RT/CT. After 3 cycles of monotherapy with TMZ and StPT, a control imaging performed in November 2014 showed the progression of the tumor (Fig. 6d). After that, the composition of the herbal medicines was modified, and PTS was introduced instead of StPT. Control imaging performed in March 2015 showed a cessation of tumor growth, while the dimensions of the tumor remained the same (Fig. 6e). In the course of the next 4 months following the control imaging, the patient’s condition was stable, with the occasional introduction of antiedema therapy with corticosteroids. In early August 2015, the patient’s condition deteriorated, she fell into a coma and died shortly after, 4 years after the initial diagnosis.

The results achieved by introducing PTS in patients 3 and 5 after the progression of the tumor, and the use of PTS in patient 4 as the first choice of treatment, show that this combination of herbal medicines may have a place in the treatment of patients with a poor prognosis. Using standard phytotherapy in the first two patients and partially in patient 5 undoubtedly contributed to their multi-year survival without the progression of the tumor. The efficacy of this combination of herbal medicine can be explained, among other things, by the low extent of cerebral edema after surgery, and it demonstrates that this herbal combination can help those suffering from GBM where cerebral edema does not obstruct the flow of pharmacologically active ingredients to tumor cells.

This research paper has shown that oncological treatment and treatment with herbal medicines are not mutually exclusive, which opens the possibility of the simultaneous use of these two methods of treatment.

The patients took PT for 24 to 48 months. During the period in which they had been taking PT, regardless of the length of treatment, the patients had no contraindications, nor did the treatment with herbal medicines caused them any problems. Researches about derivative artemisinin hepatotoxicity combined with TMZ have shown that this combination can lead to liver damage [34]. However, in these researches, they mentioned artemisinin derivative concentrations which are a lot higher than those from genus *Artemisia* L. exposed to the patients in the form of tea. The patients have been following liver markers regularly and not even once this analysis showed a more meaningful growth of the markers.

The duration of PT is not precisely defined, and the optimal time of its use remains unknown, but it is certain that it should be taken as long as there are clinical or radiological signs of a tumor. Thus, at the time of publication of this research paper, the treatment of patient no. 4 with herbal medicines had not yet been completed, even though 4 years had passed since its introduction. The final results achieved in that patient, but also in other patients whose cases are described in this paper, will be presented as part of the report on the results of the use of PT in the treatment of GBM, which will include a larger number of subjects.

This research is still in progress, and it is expected to provide more reliable data on the possibilities of using herbal medicines in the treatment of GBM and to provide answers on the possibility of combined activity of herbal remedies and oncological treatment. The course of this research has greatly been determined by the results presented in this paper, which imposed PTS as the first choice in the treatment of all the patients.

## Conclusion

The results presented in this paper suggest the possibility of introducing PT as a completely new and harmless method of treating GBM. It is quite safe to conclude that the introduction of PT as a supplementary treatment in patients undergoing oncological treatment or as monotherapy in those cases where the treatment had been completed contributes to the quality of treatment and prolongs the survival of patients. The results achieved in patients in which tumor regression occurred exclusively due to the use of herbal medicines particularly point to such a conclusion.

## Abbreviations

CT: Computed tomography
DHA: Dihydroartemisinin
GBM: Glioblastoma multiforme
IDH 1: Isocitrate dehydrogenase 1
IDH 2: Isocitrate dehydrogenase 2
MGMT: O6-methylguanine-DNA methyltransferase
NMRI: Nuclear magnetic resonance imaging
PT: Phytotherapy
PTS: Phytotherapy of salvation
RT/CT: Combined radiochemotherapy
StPT: Standard phytotherapy
TMZ: Temozolomide
WHO: World Health Organization
